# Association of depression, anxiety and menopausal-related symptoms with demographic, anthropometric and body composition indices in healthy postmenopausal women

**DOI:** 10.1186/s12905-021-01338-w

**Published:** 2021-05-07

**Authors:** Nasibeh Barghandan, Neda Dolatkhah, Fariba Eslamian, Nahal Ghafarifar, Maryam Hashemian

**Affiliations:** 1 Islamic Azad University of Ahar, Ahar, Iran; 2 Physical Medicine and Rehabilitation Research Center, Aging Research Institute, Emam Reza Hospital, Tabriz University of Medical Sciences, Golgasht, Azadi Ave., Tabriz, Iran; 3 Physical Medicine and Rehabilitation Research Center, Aging Research Institute, Tabriz University of Medical Sciences, Tabriz, Iran; 4 Department of Physical Medicine and Rehabilitation, Faculty of Medicine, Tabriz University of Medical Sciences, Tabriz, Iran; 5 Department of Biology, School of Art and Science, Utica College, Utica, NY USA

**Keywords:** Menopause, Anxiety, Depression

## Abstract

**Background:**

The termination of the menstrual cycle is correlated with a number of physiological alterations and symptoms that can negatively impact emotion and mood. We aimed to investigate the association of anxiety, depression, and menopausal related symptoms with demographic, anthropometric, and body composition indices in healthy postmenopausal women.

**Methods:**

A total of 320 menopausal women were selected randomly from referrals of health centers between January and June 2018 in Tabriz/Iran. All participants completed a demographic questionnaire. Bioelectrical impedance analysis was applied to evaluate body fat mass (BFM), soft lean mass (SLM), and lean body mass (LBM) of participants. The modified Kupperman index, Beck's depression inventory-II, and Spielberger’s state-trait anxiety inventory were applied to measure the severity of menopausal-related symptoms, the frequency, and severity of the symptoms of depression and state (SA) and trait anxiety (TA), respectively.

**Results:**

Finally, 245 postmenopausal women with age of 55.33 ± 4.48 years and body mass index (BMI) of 27.96 ± 3.22 kg/m^2^ were studied. Women with the age of 55 years and older (OR 3.928, 95% CI 1.504–10.256) and also women with mild physical activity (OR 10.104, 95% CI 3.785–26.976) had a greater possibility of having mild and moderate depression in comparison with women less than 50 years old and women with moderate and severe physical activity. Moderate and severe physical activity was correlated with a lower possibility of having medium upward, relatively severe and severe TA in comparison with participants with mild physical activity in these women (OR 0.372, 95% CI 0.151–0.917). Women with higher BMI and BFM had and more severe menopause-related symptoms (r = 0.143, p = 0.025 and r = 0.139, p = 0.030, respectively) and more severe TA symptoms (r = 0.198, p = 0.018 and r = 0.151, p = 0.021, respectively). Women with lower LBM (r =  − 0.139, p = 0.031) and lower SLM (r =  − 0.128, p = 0.047) had more severe depressive symptoms.

**Conclusion:**

Postmenopausal women with higher age and lower physical activity had a greater possibility of having mild and moderate depression. Lower physical activity was also correlated with a greater possibility of having medium upward to severe TA symptoms. Postmenopausal women with higher BMI and BFM had more severe menopause-related and TA symptoms. Women with lower LBM and SLM had more severe depressive symptoms.

## Background

Today, menopause has attracted the attention of medical investigators in most societies, especially developing countries [1], because by entering this period, glandular, physical, and psychological changes occur in women that can last several years and result in many problems in women living and daily activity [2, 3]. Menopausal age varies in women and is estimated to be around 50–52 years old on average [4]. The main consequences of menopause and menstruation stop are primarily related to estrogen deficiency and include vasomotor symptoms, genital urinary tract atrophy, osteoporosis, cardiovascular disease, cancer, cognitive decline, and sexual problems [5].

Postmenopausal women are at increased risk of central obesity [6]. The process of menopause in women caused a redistribution of body fat mass (BFM) and increased android obesity, and metabolic syndrome risk by up to 60% [7]. Several other risk factors in addition to menopause can also be effective, including multiple births, taking contraceptives, physical inactivity, and smoking and alcohol [6]. In Iran, 57% of women are obese or overweight [8]. Obesity increases the risk of coronary heart disease, hypertension, dyslipidemia, and type II diabetes, reproductive disorders, and cervical, breast, and colon malignancies [9].

With the beginning of menopause, the rate of weight gain doubles, and lean body mass (LBM) decreases, and this process continues for up to two years after the last menstrual cycle [10]. According to a study on 543 menopausal women aged 42–52 years old, the fat mass increased by about 3.4 kg and skeletal muscle mass decreased by 0.23 during the 6-year period. Additionally, follicle-stimulating hormone (FSH) changes were directly related to changes in fat mass [11].

Mental symptoms such as irritability, anger, and feelings of depression also increase around menopause. It is estimated that 26–33% of women experience their first depressive episode in the menopausal transition period [12]. Various studies show that the risk of depression increases during this period in women [13, 14].

Previous studies have pointed to the correlation of anthropometric indices with depressive symptoms to some extent. To investigate the relationship between obesity and quality of life, Heidelberg et al. [15] evaluated anthropometric and depressive symptoms of 983 postmenopausal women aged 35–74 years old. Analysis of linear models showed that there was a negative correlation between obesity and abdominal obesity with physical (not psychological) quality of life. Overweight and abdominal obesity was not significantly associated with depression and, according to the results; depressed mood exacerbated the negative correlation of obesity on the physical quality of life. Also, Jasienska et al. [16], in a study of 1156 postmenopausal women aged 45–64 years old, confirmed that high body mass index (BMI) was associated with a lower score of depressive symptoms.

The correlation of obesity with vasomotor symptoms has also been of particular interest to researchers. Earlier researchers believed that BFM protects against vasomotor symptoms due to the conversion of androgens to estrogens in adipose tissue [17, 18]. However, later studies showed that BMI [19, 20] and especially BFM [21, 22] are correlated with increased vasomotor symptom reporting. However, the correlation between anthropometric indices and body composition analysis with menopausal, anxiety, and depression symptoms in postmenopausal women has been less specifically addressed in the Iranian population. Due to the scarcity of studies and the contradictory results of previous studies, the present study aimed to investigate the correlation of depression, anxiety, and menopausal-related symptoms with demographic, anthropometric, and body composition indices in healthy postmenopausal women referred to Tabriz Health Centers in 2018. It was hypothesized that some demographic, anthropometric, and body composition indices of participants would correlate with depression, anxiety, and menopausal-related symptoms in these women.

## Methods

### Survey design

The present cross-sectional study was performed on healthy postmenopausal women referred to Tabriz Health Centers between January and June 2018. Ethical concerns of the study were approved by the Ethical Committee of the Research Vice-Chancellor and written informed consent was obtained from all participants and legally authorized representative/relatives of illiterate participants involved in the study.

### Participants and procedure

**Fig. 1:**
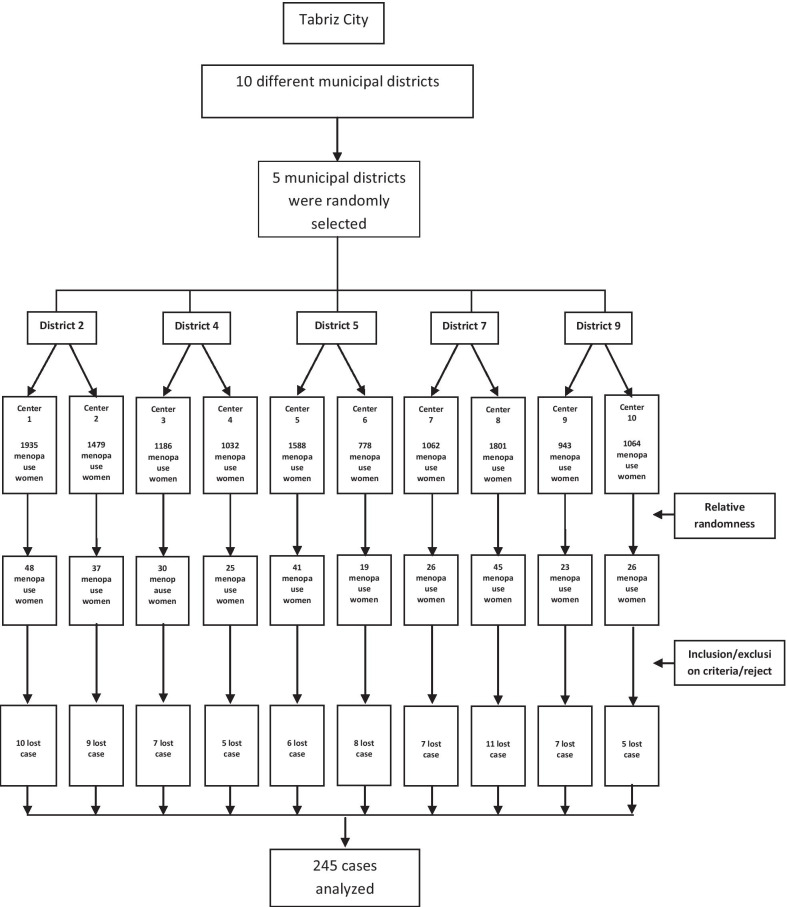
Flowchart of participants

Cluster sampling was conducted across Tabriz health centers. Tabriz has 87 health centers in 10 different municipal districts that include all postmenopausal women characteristics such as phone numbers and postal addresses in the center. Cluster-random sampling method was carried out in the health centers. Firstly, five municipal districts were randomly nominated (2, 4, 5, 7 and 9). Then, two centers in each area were randomly selected. Three trained data collectors then went to selected centers, extracted a list of all postmenopausal women aged < 65 years old, randomly selected participants through relative randomness method (the number of postmenopausal women covered by each health center) (Fig. 1), telephoned them, and after a brief explanation of the aims and method of the research, asked them to attend Tabriz Physical Medicine and Rehabilitation Research Center in due time. At the meeting, the aims of the study were fully explained and the study criteria were assessed, and if eligible, informed consent was obtained.

Inclusion criteria included healthy women aged < 65 years old, natural menopause, and one to five years past the last menstrual cycle. Exclusion criteria included chronic diseases such as diabetes, a history of cancer, a history of depression or other psychiatric diseases, taking anti-anxiety or anti-depression drugs, taking hormone-containing food supplements during the past 6 months, use of medications that affect menopausal symptoms, use of menopausal replacement therapy in the past 6 months or incomplete information.

According to the study of Zhu K et al. [23] and considering $\uprho = 0.11$, α = 0.05, β = 0.20 and using the two-sided test, the sample size was calculated to be 240 people.

### Data collection

All participants completed a demographic questionnaire by the data collectors through a face-to-face interview at the Physical Medicine and Rehabilitation Research Center of Tabriz University of Medical Sciences, Tabriz, Iran containing the following demographic characteristics: age (year), menopause age (year), marital status (married/single/other), education (illiterate/under diploma/diploma/college) and occupation (unemployed/employed/retired/housewife).

### Anthropometric measurement

Anthropometric measurements included weight (nearest 0.1 kg) by a digital scale (Seca, Hamburg, Germany) and height (nearest 0.1 cm) by non-stretched tape measure (Seca, Hamburg, Germany) examinations. Height and weight were measured without shoes and heavy clothing. From these, BMI (kg/m^2^) was calculated. The BMI was classified based on the World Health Organization (WHO) classification into normal (18.50–24.99), overweight (25.00–29.99), obese (≥ 30.00) [24].

### Body composition analysis

All participants went through body composition analysis with bioelectrical impedance analysis (BIA) using Inbody 270 (Biospace Co., Seoul, Korea) to evaluate body fat mass (BFM), soft lean mass (SLM), and lean body mass (LBM). Body composition is calculated from the alteration in conduction as fat-free body mass provides minor impedance to electrical signal because of a high amount of water and electrolytes whereas fat mass provides very low direction to electrical flows [25].

### Menopausal-related symptoms

The Persian version of the modified Kupperman index (mKMI) is extensively applied to estimate the severity of menopausal-related symptoms. It contained the following nine components: hot flashes, night sweats, insomnia, nervousness, depression, fatigue, headache, frequency, and bladder pain. Scores for every item of the modified KI ranges from 0 to 3 (0 = no symptoms, 1 = mild, 2 = moderate, 3 = severe). The weighting factors for hot flashes were four points and for night sweats, insomnia and nervousness were two points and the remaining items are left without factor. Total scores ranged from 0 to 45, with greater scores representing more severe menopausal-related symptoms. This questionnaire had been shown to have reliability and validity for recognizing menopausal-related symptoms [26, 27].

### Depression

Beck's depression inventory-II (BDI-II) is one of the most commonly used methods in research and clinical practice for assessing the frequency and severity of the symptoms of depression [28–30]. The subscales of BDI-II are classified into affective (8 items) and somatic (13 items). Each item is valued on a 4-point Likert scale ranged from 0 to 3, according to the intensity in the preceding two weeks. The total score ranged from 0 to 63, with greater scores demonstrating more severe depressive symptoms. A score of 0–13 is considered no depression, 14–19 mild, 20–28 moderate, and 29–63 is considered severe depression [30]. The internal reliability consistency of the BDI-II has been reported as 0.48–0.68 in a previous study [31].

### Anxiety

Spielberger’s state-trait anxiety inventory (STAI) is a self-administered survey, comprised of 40 items allocated into two subscales: the state anxiety (SA) subscale (1–20) and the trait anxiety (TA) subscale had 20 items (21–40) [32]. The item scoring was from 1 to 4. Positive items had scored from never (4), sometimes (3), often (2), to always (1) and negative ones had a reverse scoring from never (1), sometimes (2), often (3), to always (4). So the scores on each of the two scales vary between 20 and 80 with higher scores indicating more anxiety. The validity and reliability of the Persian version of the STAI have been established. Reliability using the Cronbach alpha coefficient was 0.91 for SA, 0.90 for TA, and 0.94 for STAI [33]. The scores were analyzed in five categories of anxiety as a state: mild (20–31), medium downward (32–42), medium upward (43–53), relatively severe (54–64), and severe (≥ 65) [34].

### Physical activity

Physical activity was estimated by the Persian version of the International Physical Activity Questionnaire (IPAQ) [35–37]. Three classifications were defined: low, moderate, and high physical activity [38].

### Data quality monitoring

All data were kept in a database generated for this study by the Physical Medicine Research Center. The data were move into database by a trained staff. Each participant had a sequential ID number. An independent data quality monitoring team was established as stated by the research validity guidelines of the research center. This team met every month to control the informed consents, adherence to the inclusion and exclusion criteria and study progress.

### Statistical methods

Statistical analysis was performed via the statistical package for the social sciences software version 17 (SPSS Inc., Chicago, IL, USA) by means of descriptive and analytic statistics. The normal distribution of numerical data was evaluated through the Kolmogorov–Smirnov test and also skewness and kurtosis. Participants were divided into three categories according to KI, BDI-II, and SA, and four categories according to TA. The correlations between demographic data and KI, and BDI, SA, and TA were assessed using Fisher’s exact test. The strength of correlation between the variables was evaluated through the odds ratio (OR) with confidence interval (CI).In addition; Spearman's correlation analysis and partial Pearson’s correlation analysis were applied to examine the correlation between body composition indices and mKI, BDI-II, SA, and TA. A p alue of less than 0.05 was considered statistically significant.

## Results

In this study, 320 postmenopausal women were randomly selected from health centers, which of them, 61 women did not accomplish the eligibility criteria and 14 women rejected to participate in the study. Finally, 245 postmenopausal women were studied to investigate the correlation between demographic characteristics, anthropometric indices, and body composition analysis with depression, anxiety, and menopausal-related symptoms.

### Participant demographics and anthropometric and body composition indices

Table 1 shows participants’ demographic and physical activity details. The mean ± SD age and menopause age presented were 55.33 ± 4.48 and 48.60 ± 4.31 years old. As to the BMI, 33 (13.5%) were normal weight, 158 (64.5%) were overweight (BMI: 25–29.9 kg/m^2^) and 55 (22.0%) were obese (BMI ≥ 30 kg/m^2^). As to the marital status, education, and occupation, 230 (93.9%) were married, 164 (66.9%) were under diploma and 206 (84.1%) were housewives. BFM, SLM, and LBM of participants were 25.63 ± 4.19 kg, 39.55 ± 5.66 kg and 43.85 ± 5.60 kg, respectively.

**Table 1:** Demographic and anthropometric characteristics of postmenopausal women

Variable	Frequency		Percent
Age (years)	55.33 ± 4.48 (Mean ± SD)		
Menopause age (years)	48.60 ± 4.31(Mean ± SD)		
Weight (kg)	69.50 ± 8.71 (Mean ± SD)		
Height (cm)	157.62 ± 4.31 (Mean ± SD)		
BMI (kg/m^2^)	27.96 ± 3.22 (Mean ± SD)		
BMI classification	18.5–24.9	33	13.5
	25–29.9	158	64.5
	≥ 30	54	22.0
Marriage	Single	9	3.7
	Married	230	93.9
	Others	6	2.4
Education	Illiterate	9	3.7
	Under Diploma	164	66.9
	Diploma	56	22.9
	College	16	6.5
Occupation	Unemployed	18	7.3
	Employed	5	2.0
	Retired	16	6.5
	Housewife	206	84.1
Physical activity	Mild	169	69.0
	Moderate	72	29.4
	Severe	4	1.6

The data are presented as Mean ± SD or frequency (percent)

### Participant depression, anxiety and menopausal-related symptom scores

Table 2 shows general findings of participants’ mKI, BDI-II and STAI scores. The mKI score, BDI-II score, SA score and TA score of participants were 27.38 ± 6.21, 21.72 ± 4.78, 41.71 ± 4.72 and 40.97 ± 7.18, respectively. According to BDI-II results, 213 (86.9%) of participants had mild depression. According to STAI results, 128 (52.2%) and 110 (44.9%) of participants had medium downward and medium upward SA, respectively, and 151 (61.6%) and 66 (26.9%), 16 (6.5%) of participants had medium downward and medium upward TA, respectively.

**Table 2:** Kupperman index, Beck depression inventory-II and State and Trait anxiety scores of postmenopausal women

Variable	Frequency	Percent
Modified Kupperman Index	27.38 ± 6.21 (Mean ± SD)	
Beck depression inventory-II	21.83 ± 4.63 (Mean ± SD)	
Beck depression inventory-II		
No depression (0–13)	26	10.6
Mild depression (14–19)	213	86.9
Moderate depression (20–28)	6	2.4
State anxiety	41.71 ± 4.72 (Mean ± SD)	
State anxiety		
Mild (≤ 31)	7	2.9
Medium downward (32–42)	128	52.2
Medium upward (43–53)	110	44.9
Trait anxiety	40.97 ± 7.18 (Mean ± SD)	
Trait anxiety		
Mild (≤ 31)	10	4.1
Medium downward (32–42)	151	61.6
Medium upward (43–53)	66	26.9
Relatively severe (54–64)	16	6.5
Severe (≥ 65)	2	0.9

The data are presented as Mean ± SD or frequency (percent)

### Participant depression, anxiety and menopausal-related symptom scores in correlation with demographic and physical activity data

The correlation of the mKI, BDI-II, and STAI with demographic and physical activity data are presented in Tables 3 and 4. There were no significant differences in participant mKI and SA scores regarding demographic and physical activity characteristics (all p > 0.05). However, there were significant differences in participant BDI-II scores concerning age and physical activity of participants. Participants with the age of 55 years and older had a greater possibility of having mild and moderate depression in comparison with participants less than 50 years old (OR 3.928, 95% CI 1.504–10.256). Furthermore, women with mild physical activity had a greater possibility of having mild and moderate depression in comparison with participants with moderate and severe physical activity (OR 10.104, 95% CI 3.785–26.976). Additionally, there were significant differences in participant TA scores considering the physical activity of participants. Women with moderate and severe physical activity had a lower possibility of having medium upward, relatively severe and severe TA in comparison with participants with mild physical activity (OR 0.372, 95% CI 0.151–0.917).

**Table 3:** Relationship between demographic characteristics and physical activity with Kupperman index and Beck depression inventory II in postmenopausal women (N = 245)*

Characteristics	Modified Kupperman Index			P*	Beck Depression Inventory II			P*
	16–25	26–35	36–45		No (0–13)	Mild (14–19)	Moderate (20–28)	
Age (years)								
< 55	45 (40.17%)	51 (45.53%)	16 (14.30%)	0.512	19 (16.96%)	92 (82.14%)	1 (0.90%)	0.004**
≥ 55	53 (46.90%)	67 (59.29%)	13 (9.19%)		7 (5.26%)	121 (90.97%)	5 (3.77%)	
Education								
Illiterate	2 (22.22%)	5 (55.56%)	2 (22.22%)	0.294	1 (11.12%)	8 (88.88%)	0 (00.00%)	0.818
Under diploma	60 (36.58%) 27	83 (50.60%)	21 (12.82%)		15 (9.20%)	144 (88.34%)	5 (2.46%)	
Diploma	(48.21%)	23 (41.07%)	6 (10.72%)		7 (12.50%)	48 (85.71%)	1 (1.799%)	
College	9 (56.25%)	7(43.75%)	0(00.0%)		3 (18.75%)	13 (81.25%)	0 (00.00%)	
Occupation								
Unemployed	7 (38.88%)	9 (50.00%)	2 (11.12%)	0.512	3 (16.66%)	14 (77.77%)	1 (5.57%)	0.514
Employed	4 (80.0%)	1 (20.0%)	0 (00.0%)		0 (00.0%)	5 (100.00%)	0 (00.00%)	
Retired	8 (50.00%)	8 (50.00%)	0 (00.00%)		3 (18.75%)	13 (81.25%)	0 (00.00%)	
Housewife	79 (38.34%)	100(48.54%)	27(13.12%)		20 (9.70%)	181(87.86)	5 (2.44%)	
Marital status								
Single	5 (55.55%)	4 (44.45%)	0 (00.0%)	0.767	1 (11.12%)	8 (88.88%)	0 (00.00%)	0.751
Married	91 (39.56%)	110 (47.82%)	29 (12.62%)		24 (10.43%)	200 (86.95%)	6 (2.62%)	
Other	2 (33.34%)	4 (66.66%)	0 (0.00%)		1 (16.67%)	5 (83.33%)	0 (00.00%)	
Physical activity								
Mild	63 (37.27%)	86 (50.88%)	20 (11.85%)	0.486	6 (3.56%)	157 (92.88%)	6 (3.56%)	< 0.001**
Moderate	34 (47.22%)	29 (40.27%)	9 (12.54%)		18 (25.00%)	54 (75.00%)	0 (00.00%)	
Severe	1 (24.00%)	3 (75.00%)	0 (00.00%)		2 (50.00%)	2 (50.00%)	0 (00.00%)	

Values are presented as n (%); (*): Using Fisher’s exact test; (**): Significant correlation

**Table 4:** Relationship between demographic characteristics and physical activity with State and Trait anxiety in postmenopausal women (N = 245)

Characteristics	State anxiety			P*	Trait anxiety					P*
	Mild (≤ 31)	Medium downward (32–42)	Medium upward (43–53)		Mild (≤ 31)	Medium downward (32–42)	Medium upward (43–53)	Relatively severe (54–64)	Severe (≥ 65)	
Age (yr)										
< 55	3 (2.73%)	64 (57.14%)	45 (40.17%)	0.368	2 (1.80%)	69 (61.60%)	33 (29.46%)	8 (7.14%)	0 (00.0%)	0.334
≥ 55	4 (3.01%)	64 (48.12%)	65 (48.87%)		8 (6.01%)	82 (61.65%)	33 (24.81%)	8 (6.01%)	2 (1.52%)	
Education										
Illiterate	0 (00.0%)	4 (44.45%)	5 (55.55%)	0.974	2 (22.22%)	5 (55.55%)	1 (11.11%)	1 (11.11%)	0 (00.0%)	0.605
Under diploma	5 (3.06%)	87 (53.04%)	72 (43.90%)		6 (3.65%)	101 (61.58%)	44 (26.82%)	11 (6.70%)	2 (1.25%)	
Diploma	1 (1.80%)	29 (51.78%)	26 (46.42%)		2 (3.59%)	33 (58.92%)	17 (30.35%)	4 (7.14%)	0 (00.0%)	
College	1 (6.25%)	8 (50.00%)	7 (43.75%)		0(00.0%)	12(75.00%)	4(25.00%)	0(00.0%)	0(00.0%)	
Occupation										
Unemployed	1 (5.57%)	12 (66.66%)	5 (27.77%)	0.247	0 (00.00%)	9 (50.00%)	6 (33.33%)	3 (16.67%)	0 (00.0%)	0.578
Employed	0 (00.0%)	4 (80.0%)	1 (20.0%)		1 (20.0%)	4 (80.0%)	0 (00.00%)	0 (00.00%)	0 (00.00%)	
Retired	1 (6.25%)	6 (37.50%)	9 (56.25%)		0 (00.00%)	12 (75.00%)	4 (25.00%)	0 (00.00%)	0 (00.00%)	
Housewife	5 (2.44%)	106(51.45)	95(46.11%)		9(4.36%)	126(61.16%)	56(27.18%)	13(6.31%)	2(0.99%)	
Marital status										
Single	1 (11.12%)	4 (44.44%)	4 (44.44%)	0.503	1 (11.11%)	7 (77.78%)	1 (11.11%)	0 (00.00%)	0 (00.00%)	0.732
Married	6 (2.62%)	121 (52.60%)	103 (44.78%)		9 (3.91%)	140 (60.86%)	63 (27.39%)	16 (6.95%)	2 (0.89%)	
Other	0 (00.00%)	3 (50.00%)	3 (50.00%)		0 (00.00%)	4 (66.67%)	2 (33.33%)	0 (00.00%)	0 (00.00%)	
Physical activity										
Mild	5 (2.96%)	88 (52.07%)	76 (44.97%)	0.423	10 (5.9%)	97 (57.40%)	52 (30.76%)	10 (5.92%)	0 (00.00%)	0.014**
Moderate	2 (2.78%)	36 (50.0%)	34 (47.22%)		0 (00.00%)	52 (72.22%)	13 (18.05%)	5 (6.94%)	2 (2.79%)	
Severe	0 (00.00%)	4 (100.00%)	0 (00.00%)		0 (00.00%)	2 (50.00%)	1 (25.00%)	1 (25.00%)	0 (00.00%)	

Values are presented as n (%); (*): Using Fisher’s exact test; (**): Significant correlation

### Participant depression, anxiety and menopausal-related symptom scores in correlation with anthropometric and body composition indices

The results of examining the correlation between mKI, BDI-II, SA, and TA with anthropometric and body composition indices are shown in Table 5. There were significant but weak positive correlations between mKI score and BMI (r = 0.143, p = 0.025) and also BFM (r = 0.139, p = 0.030). Women with higher BMI and BFM had higher mKI scores and more severe menopause-related symptoms. There were weak negative correlations between BDI-II scores and LBM (r =  − 0.139, p = 0.031) and SLM (r =  − 0.128, p = 0.047) after adjusting for physical activity and age. Women with lower LBM and SLM had higher BDI-II scores and more severe depressive symptoms. There were weak positive correlations between TA score and BMI (r = 0.198, p = 0.018) and also BFM (r = 0.151, p = 0.021). Women with higher BMI and BFM had higher TA scores and more severe trait anxiety symptoms.

**Table 5:** Relationship between body composition analysis and Kupperman index, Beck depression inventory-II and State and Trait anxiety of postmenopausal women

Variables	BMI	BFM	LBM	SLM
Kupperman Index*	r = 0.143	r = 0.139	r = 0.076	r = 0.083
	p = 0.025	p = 0.030	p = 0.234	p = 0.193
Beck depression inventory-II **	r = 0.014	r = 0.104	r = − 0.139	r = − 0.128
	p = 0.834	p = 0.108	P = 0.031	p = 0.047
State anxiety*	r = 0.111	r = 0.080	r = 0.045	r = 0.056
	p = 0.084	p = 0.210	p = 0.481	p = 0.380
Trait anxiety***	r = 0.198	r = 0.151	r = 0.091	r = 0.115
	p = 0.018	p = 0.021	p = 0.127	p = 0.079

^*^Spearman's correlation analysis, **partial Pearson’s correlation analysis adjusted for age and physical activity; ***partial Pearson’s correlation analysis adjusted for physical activity; BFM, body fat mass; BMI, body mass index; LBM, lean body mass; SLM, slim lean mass

## Discussion

The present study has investigated the correlation between depression, anxiety, and menopausal related symptoms, with demographic, anthropometric, and body composition indices in a random sample of healthy menopausal women in Tabriz city, Iran. In our study, the majority of randomly selected postmenopausal women (86.5%) were overweight or obese. Middle-aged women are at greater risk for the consequences of weight mismanagement, including obesity [39]. Since 2014, 40% of women in mid-life were classified as overweight or obese [39]. While biological mechanisms such as fluctuations in estrogen levels and changes in body fat distribution are usually associated with weight gain in middle age, psychological factors may also contribute to increasing BMI at this time.

Obese women have more symptomatic menopause than normal-weight women [40]. Studies show that there is a correlation between lifestyle (including nutrition and BMI) and the severity of menopausal symptoms [41]. Some studies have suggested that a decrease in endogenous estrogen levels may alter the amount and distribution of body fat and lead to an increase in total body fat and an increase in central fat mass in postmenopausal women [42–45]. However, some researchers suggest that the observed difference in fat mass or distribution in middle age women is largely due to the aging process and that menopausal status is either ineffective or having little effect, although there is much controversy [46–48].

In our study, there were no significant differences in participant mKI scores regarding demographic and physical activity characteristics. However menopausal-related symptoms had a very weak positive correlation with BMI and BFM in our study. That means, women with higher BMI and BFM had more severe menopausal- related symptoms, to some extent. In line with our results, in a large cross-sectional study, over 16,000 women aged 40–55 years, BMI was positively correlated with hot flashes or night sweats, urinary incontinence and joint stiffness and pain [49]. Also at the Women's Health Initiative, urogenital symptoms, including vaginal discharge, itching, and burning, were 2–4 times higher in obese women than in normal-weight women [50]. This is while some other studies did not show a correlation between BMI and vasomotor symptoms in postmenopausal women [51–54].

In our study, 86.9% of participants had moderate depression symptoms and only 10.6% of participants didn’t present depression symptoms. Despite the considerable burden that menopausal depression has on millions of women, little is known about its underlying biological mechanisms. A recent review article comprised 12 cross-sectional studies comparing the prevalence of depression symptoms in pre and peri-menopausal women and found that 45–68% of peri-menopausal women compared to only 28–31% of pre-menopausal women report a significant increase in clinical symptoms of depression [55]. Depression is prevalent in this population, with menopause being listed as a "Window of vulnerability" to develop depressive symptoms that are partially justified by changes in hormone levels and lifestyle factors [14, 56, 57]. A prompt process of aging has happened in the past decades in Iran's population [58]. Elderlies face problems such as joblessness, loneliness, and decreased income that have a deleterious effect on the quality of life and mental health and older postmenopausal women are more likely to be involved by these problems [59], which, at least in part, explain the high prevalence of depression in studied menopause women.

However, less is known about anthropometric changes and body composition affecting perimenopausal depressive symptoms. While depression leads to poor weight outcomes in different populations, it is important to examine this association in middle-aged and postmenopausal women given the high prevalence of both conditions in this population [60, 61]. Controversy continues in the medical literature about the correlation between the severity of symptoms of depression and anxiety and anthropometric indices. Some studies have suggested weight gain in itself as an important determinant of psychological symptoms [62, 63]. In other studies, however, fat distribution has been the most dominant factor [64, 65].

On the other hand, psychiatric disorders are likely to interfere with physical mass distribution, especially by facilitating the storage of visceral fat. Depression and the consequences of weight management in peri and post-menopause women make them at higher risk of depression and obesity than non-menopausal women [16]. The reasons for this increase are due to a combination of hormonal changes and environmental stress [66]. In our study, there were significant correlations between BDI-II score and age and physical activity. That means participants with higher age and lower physical activity had a greater possibility of having mild and moderate depression. Lifestyle characteristics such as physical activity have been identified as a contributing factor to depressive symptoms. Lack of physical activity is one of the effective factors in causing obesity, especially in old age and menopause, so that according to studies conducted in Iran, more than 40% of adult Iranians, especially women, have little physical activity [67]. Consistent with the results of the present study, Sternfeld et al. [68] confirmed that 12 weeks of moderate-intensity aerobic exercise had no effect on menopausal vasomotor symptoms but slightly improved sleep quality as well as reduced insomnia and depression in the sedentary middle-aged women. However, other studies have emphasized the positive role of physical activity in reducing hot flashes and other symptoms of menopausal syndrome [69, 70].

In our study, there was no significant correlation between participant BDI-II scores and BMI also BFM. However, there was a very weak negative correlation between the BDI-II score, and LBM and women with lower BFM showed a higher score in BDI-II and more severe depression symptoms.

During the period of the menopausal process, the mean body fat gain doubles in an average woman from approximately 1–1.7%, and resulted in a 6% increase in total body fat over the 3.5 year period (an average total weight gain of 1.6 kg). As menopause initiates, women begin to lose lean mass. The total loss of lean mass during the menopause transition is 0.5% on average (mean decrease of 0.2 kg) [10]. Particular essential factors such as physical inactivity, protein intake and oxidative stress contribute to sarcopenia in postmenopausal women [71–73]. There are several reports showing that anthropometric indices and biomarkers of central and generalized obesity, correlate with different aspects of psychological health in the general population [74–76]. In line with our results, in the study of Cugini et al. [77], depression was established to be correlated, negatively, with the relative LBM in obese participants but not in clinically healthy subjects. Additionally, Schreiber et al. [78] examined the correlation between depressive symptoms and weight using information from the Midlife in the United States II study and found that depressive symptoms were not directly correlated with weight. However, stress eating was a significant mediator between depressive symptoms and weight. Because depression is usually associated with increased calorie intake, a potential mechanism that relates symptoms of depression and weight gain is eating behaviors. Some eating behaviors, are associated with mood swings and depressive symptoms and are also known to be a risk factor for obesity [79, 80]. On the other hand, overweight/obesity can weaken self-esteem and sexual and social health, and disrupt psychological wellbeing [81, 82].

According to our findings, there were weak correlations between participant TA scores and physical activity. That means women with mild physical activity had a higher possibility of having medium upward, relatively severe and severe TA in comparison with women with moderate and severe physical activity. Furthermore, there were weak positive correlations between participant TA scores and BMI and also BFM. That means women with higher BMI and BFM have more severe TA symptoms after adjusting for physical activity. The bidirectional correlation between anxiety and obesity has been less examined. Numerous factors could elucidate the relationship between obesity and anxiety [83, 84], one of which is immune-inflammatory stimulation, as both conditions increase inflammatory biomarkers, such as C-reactive protein (CRP), interleukin-6 (IL-6) and tumour necrosis factor-alpha (TNF-α) [83]. Several adjustable lifestyles, such as lower physical activity and unhealthy dietary patterns, are correlated with an increased risk of both obesity and depression/anxiety [83, 84]. On the other hand, weight perception, lesser social support, and social networks could augment the risk of anxiety in obese persons [82]. Pressuring people to lose weight can be stressful for obese individuals and raise the risk of anxiety, especially when several efforts to lose weight have been unsuccessful [82]. Anxiety symptoms may rise appetite and the tendency for comfort foods and therefore, result in obesity [85].

The results of the present study add to the extensive knowledge available on the relationship between anthropometric and body composition indices with menopausal-related symptoms and depression and anxiety in postmenopausal women. This study provides novel results regarding these relationships. To our knowledge, no other study has examined the relationship in this way, so future studies may help confirm or reject the present results.

However, this study has some limitations that should be considered in interpreting the results. The cross-sectional design of the study precluded the establishment of a casual or pathophysiologic relationship. In addition, the sample size is relatively small. Furthermore, some estimations of the correlation coefficient are small suggesting weak correlation in spite of statistically significant p-values. Additionally, the provided ORs are not very reliable because of the small sample size of categories. Finally, despite investigating multiple variables, residual confounders and missing data are always among the limitations of observational studies.

## Conclusions

In conclusion, our study confirmed that postmenopausal women with higher age and lower physical activity had a greater possibility of having mild and moderate depression. Lower physical activity was also correlated with a greater possibility of having medium upward to severe trait anxiety symptoms in these women. Postmenopausal women with higher BMI and BFM had more severe menopause-related and trait anxiety symptoms. Additionally, women with lower LBM and SLM had more severe depressive symptoms.

## Data Availability

All the necessary data are presented herewith. However if needed, raw data on excel format can be availed on reasonable request from the corresponding author.

## Abbreviations

BDI-II: Beck's depression inventory-II
BFM: Body fat mass
BMI: Body mass index
FSH: Follicle-stimulating hormone
KI: Kupperman index
LBM: Lean body mass
SLM: Soft lean mass
SPSS: Statistical package for the social Sciences software
STAI: Spielberger’s state-trait anxiety inventory
